# The longitudinal trajectory of body mass index in the Chinese population: A latent growth curve analysis

**DOI:** 10.1371/journal.pone.0207845

**Published:** 2018-11-26

**Authors:** Feifei Huang, Minqiang Zhang, Yan Li, Zhe Li, Junyan Fang, Kaiyin Guo

**Affiliations:** 1 School of Psychology, South China Normal University, Guangzhou, China; 2 Guangdong Key Laboratory of Mental Health and Cognitive Science, South China Normal University, Guangzhou, China; Old Dominion University, UNITED STATES

## Abstract

**Aims:**

The objective of this study was to investigate the longitudinal trajectory of BMI in the Chinese population, and to assess whether the gender or other personal characteristics were related to BMI, and whether there was a change in trajectory over time.

**Methods:**

Data were obtained from 3,574 Chinese (5 to 89 years of age) who participated in the 2000–2011 of the China Health and Nutrition Survey. Latent growth curve models were used to investigate the longitudinal trajectory of BMI, and to examine the effect of some personal characteristics on BMI trajectory.

**Results:**

The linear model resulted in a mean initial BMI value of 22.90 and a significant mean slope (*Ms* = 0.19, *t* = 10.73, *p*<0.001), suggesting a steady increase in BMI over time for the whole sample. For covariates, the educational level, alcohol and physical activity had differences on initial scores for BMI (*β* = 0.05, *p*<0.001; *β* = -0.12, *p*<0.05; *β* = -0.08, *p*<0.05; respectively.), and the age had differences on both the initial scores and slope for BMI (*β* = 0.01, *p* = <0.05; *β* = -0.03, *p*<0.01; respectively.). Baseline measures revealed gender-associated differences on initial scores for BMI, and the slope for male was significantly steeper than that for female (*β* = -0.11, *p*<0.05). The initial BMI status of Chinese living in the rural areas was significantly higher than that of Chinese living in the urban areas, and the slope for rural status was significantly steeper than that for urban status (*β* = 0.21, *p*<0.001).

**Conclusions:**

Results indicated a linear trajectory of BMI in the Chinese population over a 12-year period. The longitudinal trajectories differed by age, gender and urban-rural status, suggesting different interventions should be adopted for different groups.

## Introduction

Body Mass Index (BMI) is universally used to measure the degree of obesity and is often collected in nutrition and health surveys. However, numerous studies used the BMI cutoffs as an international criterion to measure individual overweight status recommended by the World Health Organization (WHO) [1–6]. Therefore, the change of BMI was usually studied in an oversimplified static way. In this paper, we investigate the longitudinal trajectory of BMI; we further examine whether the gender or other personal characteristics are related to BMI, and whether there is a change in trajectory over time.

BMI has achieved universally acceptance as a standard approach to diagnose obesity. The WHO recommends using BMI cutoffs of 25 and 30 kg/m^2^ to define overweight and obesity, respectively. However, it is currently controversial that whether the optimal BMI cutoffs are suitable to be applied to Chinese [7–11]. Moreover, previous studies of BMI in the Chinese population were often at a certain time point, regardless the dynamic change of BMI. To counteract this limitation, a dynamic statistical approach of latent growth curve model (LGCM) which is conducted in longitudinal designs of 3 or more waves of assessment has been developed and now used regularly. Compared with other conventional methods, LGCM has several advantages in longitudinal studies [12–15]. For example, it can provide a more complete picture of the change process because LGCM can estimate a wide range of parameters of change [12, 16]. As a family of structural equation modeling, LGCM provides statistical advantages in estimating longitudinal patterns in outcome measures [17].

It was well established that BMI was associated with gender, urban-rural status and other personal characteristics. Studies have shown the gender differences in the BMI [1, 6, 18]. In addition, higher BMI was found in rural regions in China with the economic development [6, 19]. However, we have little reliable evidence of whether the gender or other personal characteristics had differences in the longitudinal trajectory of BMI.

In this paper, we aimed to investigate the longitudinal trajectory of BMI in the Chinese population, and to assess whether the gender or other personal characteristics were related to BMI.

## Method

### Ethics statement

The CHNS project was approved by the office of human research ethics of the University of North Carolina at Chapel Hill and the Human & Clinical Research Ethics Committee of China-Japan Friendship Hospital. All participants provided written informed consent to participate in this study. The Institutional Review Board information can also be found at http://www.cpc.unc.edu/projects/china.

### Sample

The data used in this study were derived from five waves of the China Health and Nutrition Survey (CHNS), collected in 2000, 2004, 2006, 2009 and 2011. The CHNS, a nationwide longitudinal survey collected data between 1989 and 2011 in nine Chinese provinces (Guangxi, Guizhou, Heilongjiang, Henan, Hubei, Hunan, Jiangsu, Liaoning and Shandong) and three mega-cities (only in 2011), was conducted by both the Carolina Population Center and the National Institute for Nutrition and Health. As a comprehensive study, CHNS provided a fair representation of urban and rural areas using a multistage and random cluster sampling design [20]. Data are available from the China Health and Nutrition Survey (http://www.cpc.unc.edu/projects/china/ data/datasets). In this study, we excluded sample loss and missing values on our outcome variables. The longitudinal cohort study contained a total of 3,574 participants, aged from 5 to 89, 1,550 were male, and 2024 were female.

### Measurements

The main variables measured were the participants’ age, gender, urban–rural status, educational level, smoking, alcohol, physical activity, weight, and height. The baseline demographic features were collected by direct interviews [21].

Weight of participants was measured with light clothing to the nearest 0.1kg on a beam balance scale. Height of participants was measured without shoes to the nearest 0.1cm with a portable stadio-meter [20–23]. BMI was calculated as weight (kg) divided by height squared (m^2^). In this study, we used BMI as a continuous variable.

### Statistical analysis

Firstly, descriptive statistics were calculated for the individual demographic variables in each wave of the survey. Continuous variables were expressed as means and standard deviation (SD). Categorical variables were presented as percentages.

Secondly, the LGCM was analyzed to illustrate the longitudinal trajectories of BMI and examine whether individuals have different initial levels and longitudinal changes in BMI. For an unconditional model of BMI, this study estimated a two latent factors model across five waves. The first factor (intercept) was defined by fixing all five parameters of the loadings to 1.0, which represented no growth of the initial level across five waves. Loading for the second factor (slope) was fixed at values of 0, 1, 2, 3, and 4. The overall fit indices of the BMI trajectory model should be interpreted prior to reporting the results of LGCM. According to Hu and Bentler’s (1999) research [24], to test the adequacy of the research model, the values of comparative fit index (*CFI*) and Tucker and Lewis index (*TLI*) should be larger than .95, and the root mean square error of approximation (*RMSEA*) value should be below .06.

Finally, we incorporated specific covariates in a conditional LGCM, including age, gender, urban–rural status, educational level, smoking, alcohol, and physical activity, so we could analyze the impact of time-invariant baseline covariates on BMI trajectories.

Latent growth curve models were conducted using Mplus 6.1 software [25], and we used SPSS 17.0 (IBM SPSS Statistics, Armonk, USA) to conduct descriptive analysis.

## Results

### Descriptive statistics

The overall study population for the baseline survey consisted of 3,574 respondents (1550 males and 2024 females).Mean age was 46.08 years old for males and 45.94 years old for females. About 70% and 72% of males and females were rural status respectively. Descriptive statistics of the other measures were reported in Table 1.

**Table 1:** Descriptive statistics for the measures.

		Male (*n* = 1550)	Female (*n* = 2024)
**Age (M±SD)**		46.08±12.97	45.94±12.46
**Rural-urban** **status (%)**	**rural**	70.26%	71.79%
	**urban**	29.74%	28.21%
**Educational level (%)**	**none** **graduate from primary school** **lower middle school degree** **upper middle school degree** **technical or vocational degree** **university or college degree** **Master's degree or higher**	27.87% 20.32% 29.35% 12.58% 5.16% 4.58% 0.14%	27.52% 20.26% 28.36% 12.80% 6.08% 4.79% 0.20%
**Smoking** **(% yes)**		29.48%	30.73%
**Alcohol** **(% yes)**		35.16%	35.47%
**Physical activity** **(% yes)**		5.74%	6.03%
**BMI(M±SD)**			
**2000**		22.68±3.14	23.00±3.26
**2004**		22.96±3.26	23.29±3.39
**2006**		23.01±3.25	23.42±4.16
**2009**		23.30±3.34	23.53±3.45
**2011**		23.85±6.31	23.87±4.15

### Unconditional LGCM

The analysis of BMI trajectory was conducted within a latent variable framework. LGCM produced an acceptable chi-square test statistic, χ^2^ = 127.71, *df* = 10, and appropriate fit indices, *CFI* = 0.99, *TLI* = 0.99, *RMSEA* = 0.06. The linear model resulted in a mean initial BMI value of 22.90 and a significant mean slope (*Ms* = 0.19, *t* = 10.73, *p*<0.001), suggesting a steady increase in BMI over time for the whole sample (as shown in Table 2). The *Ms* value of 0.19 could be interpreted as the average increase of BMI per unit of time. The variances for the intercept and slope were 8.97 (*t* = 36.91, *p*<0.001), and 0.16 (*t* = 10.84, *p*<0.001), respectively, indicating substantial variation across individuals of initial BMI and significant change rates for BMI due to individual variability. There was no significant correlation between initial BMI and change rates (*t* = -0.75, *p*>0.05).

**Table 2:** Parameter estimates based on latent growth curve model.

Parameter	Estimate	S.E.	*t*	Standard estimate
Mean				
Intercept	22.90***	0.05	428.228	7.65
Slope	0.19***	0.01	16.316	0.47
Variance				
Intercept	8.97***	0.24	36.91	1.00
Slope	0.16***	0.02	10.84	1.00
Covariance				
Intercept < >	-0.03	0.04	-0.75	-0.03
Slope				
Factor loading				
2000	0.00	0.00	—	0.00
2004	1.00	0.00	—	0.40
2006	2.00	0.00	—	0.80
2009	3.00	0.00	—	1.20
2011	4.00	0.00	—	1.60

***p<0.001

### Conditional LGCM with covariates

We conducted a conditional LGCM analysis to examine whether covariates were related to BMI and whether there was a change in trajectory over time. The categorical covariates were coded as: (1) Gender (male = 1, female = 2); (2) Urban-rural status (rural = 1, urban = 2); (3) Educational level (none = 0, graduate from primary school = 1, lower middle school degree = 2, upper middle school degree = 3, technical or vocational degree = 4, university or college degree = 5, Master's degree or higher = 6); (4) Smoking (not = 0, do = 1); (5) Alcohol (not = 0, do = 1); (6)Physical activity (not = 0, having = 1). The analysis produced an acceptable chi-square test statistic, χ^2^ = 156.45, *df* = 31, and appropriate fit indices, *CFI* = 0.99, *TLI* = 0.99, *RMSEA* = 0.03.

**Fig 1:**
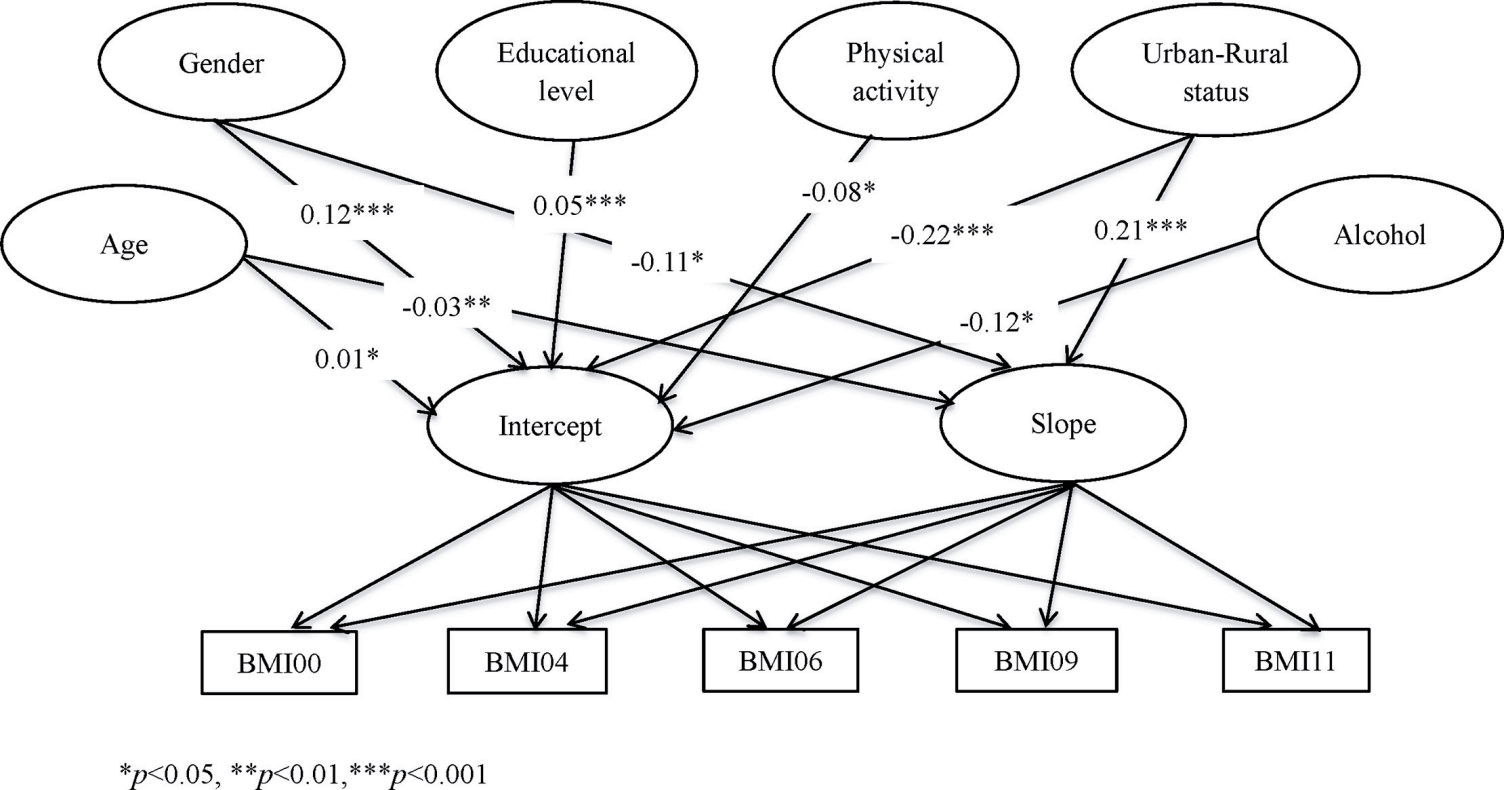
Path diagram for LGCM with covariates in the Chinese population from 2000 to 2011.

Fig 1 represented the standardized solution for the conditional LGCM with covariates. Firstly, the educational level, alcohol and physical activity had differences on initial scores for BMI (*β* = 0.05, *p*<0.001; *β* = -0.12, *p*<0.05; *β* = -0.08, *p*<0.05; respectively.), but they had no difference on the slope for BMI (*β* = -0.02, *p* = 0.44>0.05; *β* = 0.01, *p* = 0.83>0.05; *β* = 0.09, *p* = 0.14>0.05; respectively.). Secondly, the smoking had no effect on both the initial scores and the slope for BMI (*β* = 0.05, *p* = 0.27>0.05; *β* = 0.00, *p* = 0.95>0.05; respectively.). Then, the age had differences on both the initial scores and slope for BMI (*β* = 0.01, *p* = <0.05; *β* = -0.03, *p*<0.01; respectively.). What’s more, baseline measures revealed gender-associated differences on initial scores for BMI (male = 23.41, female = 23.16), and the slope for male was significantly steeper than that for female (*β* = -0.11, *p*<0.05). Finally, the initial BMI status of Chinese living in the rural areas was significantly higher than that of Chinese living in the urban areas (rural = 23.64, urban = 23.17), and the slope for rural status was significantly steeper than that for urban status (*β* = 0.21, *p*<0.001).

## Discussion

The main purpose of this study was to investigate the longitudinal trajectory of BMI by using LGCM. Besides, by including covariates, this study examined whether age, gender, urban–rural status, educational level, smoking, alcohol, physical and activity were related to BMI and whether there was a change in trajectory over time. In this study, we found that there was clearly an increase in the rate of change in Chinese’s BMI. Moreover, results showed that changes in BMI were significantly higher among male, younger cohorts and individuals with rural status. We now consider the implications of our results for the trajectory of BMI.

The results revealed a linear trajectory of BMI and the BMI of Chinese changed very little over a 12-year period. With the rapid economic growth of China since 2000s, studies have demonstrated a faster rate of increase in overweight because of overconsumption of dietary of fat [26–27]. In addition, some researchers have found that the obese reported much more eating for psychological reasons such as depression or boredom [28–30]. Psychological motivations which undoubtedly contributed to the increase in body weight are also thought to be important factors in conjunction with BMI [11]. Therefore, Chinese improve their awareness of health with the repeated public health messages about the dangers of being higher BMI in recent years.

For baseline covariates, we found that the educational level, alcohol and physical activity had differences on initial scores for BMI, but they had no difference on the slope for BMI. The smoking had no effect on both the initial scores and the slope for BMI. One of the main reasons could be that, with the spread of basic education, people tend to focus on intervention programs designed to reduce BMI through lifestyle modification in China. Therefore, such healthy context helps people to raise the awareness of their own health in China.

Consistent with previous studies, the results showed a faster rate of increase in BMI of younger than older [22, 31–33]. Researchers have found the problem of overweight among Chinese adults, suggesting that younger cohorts in China were more likely to adopt a diet with high fat and low dietary fiber [31–34]. The unhealthy behavior may result in rapid increases in the prevalence of obesity in the younger cohorts. Thus, overweight in the younger cohorts should no longer be regarded as variations of normality [35]. We should make efforts to promote Chinese adults to adopt healthy diet and lifestyle.

As indicated in the present study, the initial BMI status of men was higher than that of women during 2000 and 2011, and the results showed a faster rate of increase in BMI of men than women. Researchers demonstrated that unhealthy diet such as take-away and fast food was associated with higher BMI in men [36]. Moreover, studies have shown that men tend to eat more meat and reduce vegetable and fruit consumption when they had meals outside [20]. Therefore, higher amounts eating which is linked with weight gain will become a problem as their activity levels decrease. Besides, women have greater concern about their body weight than men because body image is an important factor to Chinese women. Hence, different health interventions should be developed for women and for men. For example, we should encourage men to decrease the frequency of away-from-home food consumption [20].

We note that our finding of rural Chinese not only had higher initial BMI status, but also their BMI increased more than urban Chinese. With the rapid development of economy and urbanization in rural areas over the recent years in China, rural regions have remarkable changes in lifestyle compared with urban regions. Previous studies have shown that there is a greater BMI in developing regions largely because of higher dietary energy-dense intake, less farming work and more sedentary time owing to the economic growth [37]^.^ However, urbanization will continue unabated due to economic disparities in China, the phenomenon of rural-urban status differences in BMI is unlikely to reverse by itself, which also indicates that the higher BMI of people will appear to increase more rapidly in the rural regions [20]. In addition, to explain why the urban-rural status difference in the longitudinal trajectory of BMI, we need to consider ethnic differences. There are many ethnic subgroups in rural areas in China. Researchers have found that genetically determined differences in body composition, metabolic response, lifestyle as well as environment and socio-economic factors may contribute to the increase in body weight [38–40]. It is possible that obesity-related diseases will become a major health problem in the rural regions. Thus, separate health interventions should be developed for rural Chinese and for urban Chinese. For example, we should encourage rural Chinese to participate in leisure time physical activity [20].

This study is meaningful because it is the first study to use LGCM to analyze the change of BMI in Chinese. Other researchers have shown the change of BMI among Chinese subjects, where most examined the trends by applying the conventional methods [18, 20–21]. Besides, the study was based on large and representative samples from China, which ensured the generalizability of the findings. Nevertheless, it is also important to bear in mind that there are some limitations in the present study. On one hand, there are potential ethnic variations in the longitudinal trajectory of BMI, but participants in our study may not be representative of the multi-ethnic Chinese population. On the other hand, we used the baseline covariates to analyze the impact of time-invariant covariates on BMI trajectories; thus we may have lost the important information. Future studies are needed to assess the impact of time-varying covariates on BMI trajectories.

In summary, we have showed a linear trajectory of BMI in the Chinese population over a 12-year period. We have also found that the longitudinal trajectories of BMI differed by age, gender and urban-rural status. As such, we have proposed that different interventions including healthy dietary behavior, education about the nutrition and physical exercises should be adopted for different groups [41]. What’s more, we have emphasized the importance of earlier prevention of overweight and obesity is needed to prevent future related problems in the Chinese population.
